# Conformational Change of Transcription Factors from Search to Specific Binding: A *lac* Repressor Case Study

**DOI:** 10.1021/acs.jpcb.2c05006

**Published:** 2022-11-23

**Authors:** Malin Lüking, Johan Elf, Yaakov Levy

**Affiliations:** † Department of Cell- and Molecular Biology-ICM, Uppsala University, Uppsala, Uppsala County751 24, Sweden; ‡ Department of Chemical and Structural Biology, Weizmann Institute of Science, Rehovot, Central District76100, Israel

## Abstract

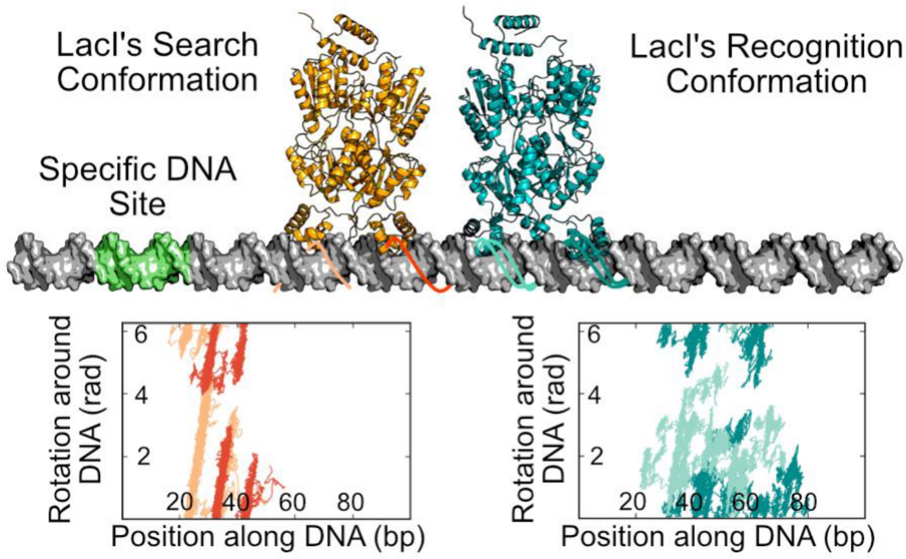

In a process known as facilitated diffusion, DNA-binding proteins find their target sites by combining three-dimensional diffusion and one-dimensional scanning of the DNA. Following the trade-off between speed and stability, agile exploration of DNA requires loose binding, whereas, at the DNA target site, the searching protein needs to establish tight interactions with the DNA. To enable both efficient search and stable binding, DNA-binding proteins and DNA often switch conformations upon recognition. Here, we study the one-dimensional diffusion and DNA binding of the dimeric *lac* repressor (LacI), which was reported to adopt two different conformations when binding different conformations of DNA. Using coarse-grained molecular dynamic simulations, we studied the diffusion and the sequence-specific binding of these conformations of LacI, as well as their truncated or monomeric variants, with two DNA conformations: straight and bent. The simulations were compared to experimental observables. This study supports that linear diffusion along DNA combines tight rotation-coupled groove tracking and rotation-decoupled hopping, where the protein briefly dissociates and reassociates just a few base pairs away. Tight groove tracking is crucial for target-site recognition, while hopping speeds up the overall search process. We investigated the diffusion of different LacI conformations on DNA and show how the flexibility of LacI’s hinge regions ensures agility on DNA as well as faithful groove tracking. If the hinge regions instead form α-helices at the protein–DNA interface, tight groove tracking is not possible. On the contrary, the helical hinge region is essential for tight binding to bent, specific DNA, for the formation of the specific complex. Based on our study of different encounter complexes, we argue that the conformational change in LacI and DNA bending are somewhat coupled. Our findings underline the importance of two distinct protein conformations for facilitated diffusion and specific binding, respectively.

## Introduction

**Figure 1:**
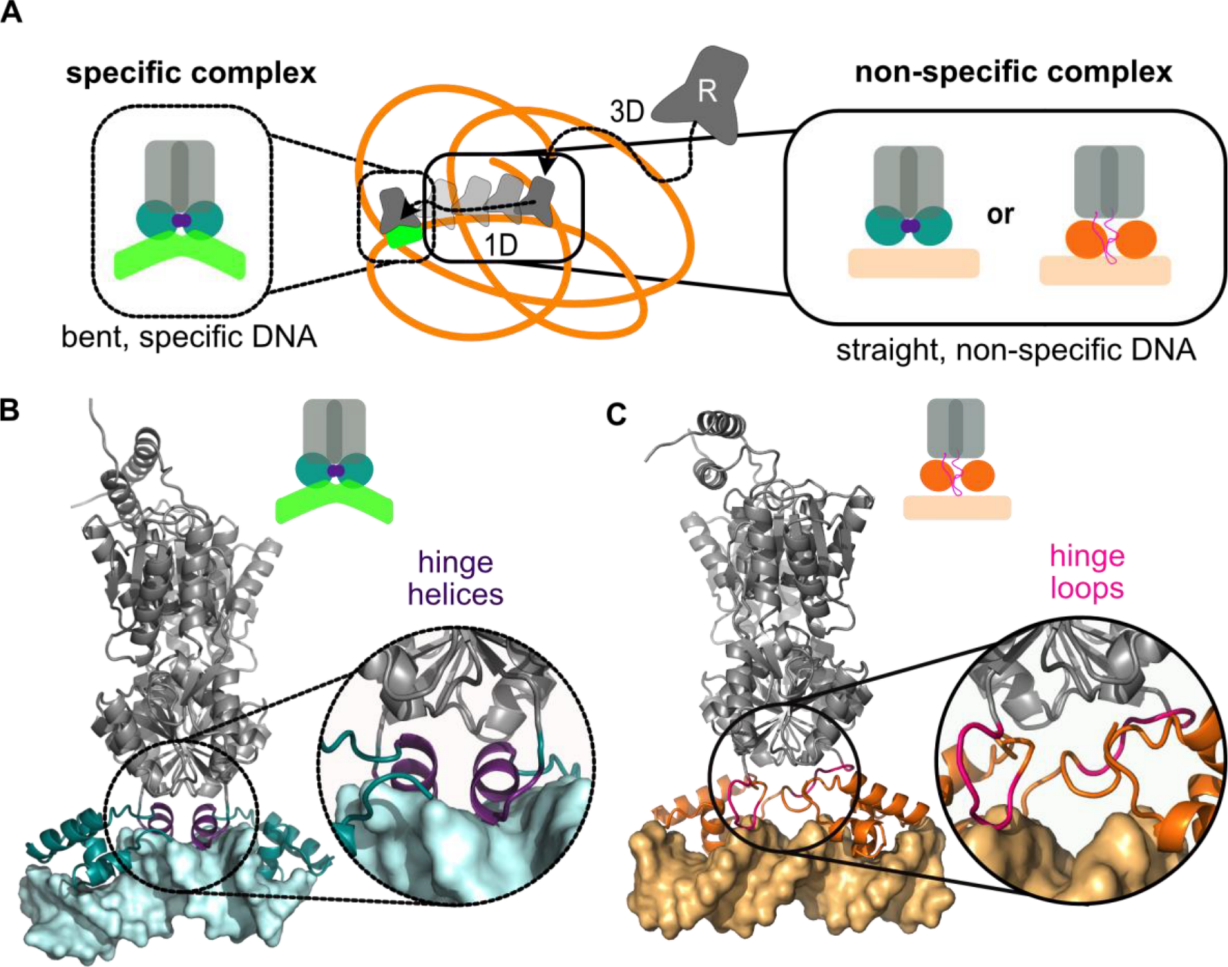
Schematic representations of the facilitated diffusion performed by LacI and the protein conformations involved. (A) Facilitated diffusion as a combination of 3D and 1D search mechanisms. The zoom panels demonstrate the structural properties of the specific and nonspecific complexes of LacI and the interacting DNA. The specific complex of LacI contains a bent DNA target as well as helices at the protein–DNA interface. The conformation of LacI in the nonspecific complex during the search process is still elusive, and usage of both conformations of LacI (or others) are possible. (B) Cartoon representation of the recognition conformation with cyan DBD and DNA, and violet, helical hinge region (PDB ID 1EFA). (C) Cartoon representation of the repressor search conformation with DBD and DNA colored in orange (PDB ID 1OSL) and the hinge region in pink. In both conformations, the core domain is shown in gray. The full-length search conformation was modeled by incorporating the core domain adopted from the full-length structure of the recognition conformation.

DNA binding proteins (DBPs) can quickly find and bind their target sites in a vast space of potential binding sites by facilitated diffusion.^1−3^ The “facilitated-diffusion” model proposes that DBPs randomly bind to nontarget DNA and approach their respective target sites while interchanging between one-dimensional (1D) diffusion and free three-dimensional (3D) diffusion (Figure 1A).^1,3^ The rates for specific binding show a clear dependency on the salt concentration,^4−6^ leading to the understanding that 1D diffusion is maintained by electrostatic interactions between positively charged protein residues and the negatively charged DNA backbone. The 1D diffusion can be performed in different modes of translation and be modulated by external factors such as salt concentration. On-dimensional diffusion of DBPs along DNA can be rotation-coupled,^7,8^ likely guided by the geometry of the DNA major groove. Here, we will call this translational mode groove tracking, but this motion is also often referred to as sliding.^9^ We use groove tracking following the experimental study on the same system.^8^

It is still debated how proteins recognize their specific sites,^10^ but energetically, a lowered barrier for establishing tight interactions with DNA is supposedly a marker for it.^11^ This lowered barrier might be caused by compatible hydrogen bond donor and acceptor patterns between the interacting molecules, or favorable interactions that are established based on the compatibility of the DNA’s and the protein’s respective shapes at the target site while the protein is helically tracking the grooves.^10,12^ While it is likely important for specificity, groove tracking is inefficient for the exploration of many sites because it is redundant and locally traps the protein at nonspecific sites.^13^ In an alternative mode of translation, DBPs may diffuse along the DNA by a mechanism that is not rotationally coupled to translation. We refer to this mode as hopping.^9^ When hopping, the DBP dissociates from and reassociates with the DNA at a relatively close site, usually 10 or multiples of 10 base pairs (bp) away from the site of dissociation.^1,14−17^ In this mode of translation, the DBP is not committed to the major groove and is not as tightly interacting with the DNA which results in faster diffusion.^18^ If one describes 1D diffusion on DNA as diffusion in a rough potential, sequence-dependent variations in roughness can play a role^19^ and flanking degenerate consensus sequences may even serve as antennas that retain DBPs close to the specific site.^20^ But generally, each base-pair step during groove tracking is associated with a barrier of 1–2 kcal·mol^–1^.^21^ Initialization of hopping has a higher barrier but results in faster 1D diffusion since it moves the DBP a considerably longer distance per event.^8^

The hopping mechanism is not the only way via which the protein evades being trapped at nonspecific sites. Frequent detachment from the DNA followed by 3D diffusion that leaves the protein to relocate to other DNA sites is another possibility.^19,22^ Also, there may be different conformations of the DBP that minimize the hydrogen bond formation with the DNA during facilitated diffusion which results in a less energetically rugged energy landscape for groove tracking.^23,24^ The kinetics of protein–DNA recognition becomes much more complex when such conformational changes are involved, but they are likely crucial for the fast exploration of the DNA that still allows specific protein–DNA recognition.^25^ These conformational changes can concern either or both DBP and DNA.^26,27^ In the case of DBPs, a “two-state model” for DNA binding has been proposed. This model rests upon two distinct conformational states of the DBP: one for search, governed by electrostatic, delocalized, and loose interactions (the search conformation), and the other for specific binding, in which the DBP tightly interacts with the DNA target site via hydrogen bonds and van der Waals interactions (the recognition conformation).^1,23,28,29^ Such a two-state model for protein–DNA binding has been used to interpret the different conformations of various DBPs that were found when comparing their binding to nonspecific and specific DNA sites.^30−32^ Another aspect further complicates this model of protein DNA recognition, namely DNA conformational changes. Conformational transitions of the DNA are crucial to consider when studying protein–DNA recognition because of the various DNA conformations found in specific protein–DNA complexes such as those of the TATA-box binding protein^33^ or the *Eco*RV endonuclease.^30^ Because the DNA conformation seems to play a role in the recognition process, it likely also influences the search kinetics.^34^ Overall, we need to consider the conformational changes in protein and DNA and the coupling between them during the recognition process.^28,35^ The kinetics of these conformational transitions are crucial for understanding not only recognition but also the overall search kinetics.^24^ If we want to understand the search kinetics of DNA-binding proteins, we therefore have to understand their search and recognition conformations and their potential energy landscapes in interaction with the DNA. In fact, the relationship between conformational switching in DBPs and their search kinetics has been used for designing DBPs for optimal protein–DNA binding kinetics.^28^

In the case of the transcription factor (TF) LacI, repressor of the *lac* operon in *E. coli*, it has long been hypothesized, but never shown, that the two conformational states interchange by a folding mechanism involving the hinge region.^36−39^ The hinge region is located between the DNA and the protein’s core regions and forms the dimerization interface in the DNA-binding domain (DBD) of LacI. As shown in Figure 1B,C, the hinge regions adopt a helical conformation when LacI interacts with the specific operator,^40,41^ in the recognition conformation, whereas it is supposedly unstructured in the unbound state, the search conformation of LacI.^42^ According to NMR data of the dimeric DBD, the hinge region of the nonspecifically bound LacI is similarly flexible as in the unbound LacI,^32^ where it exhibits partially unfolded hinge helices that come with a large conformational space of the two DNA-binding domains.^43^ Here, we address the effect of the conformation of the hinge region on DNA search efficiency and on tight binding by modeling the molecular mechanisms of facilitated diffusion and recognition for the dimeric *lac* repressor with a coarse-grained (CG) molecular model of the recognition and search conformations of LacI.

## Methods

### Coarse-Grained Model

A native structure based, coarse-grained molecular model has been applied widely to study diffusion of proteins on DNA as well as on microtubules.^28,44−47^ We use a flavor of the CG model where the protein is modeled by replacing the amino acids with a unified atom model with one bead located at the Cα position and run molecular dynamics (MD) simulations. The DNA is represented by three beads per nucleotide, positioned at the geometric centers of the sugar, the base, and the phosphate groups, respectively. The native structure based coarse-grained force field is shown in eqs 1–5. The harmonic potentials of the bonded term have force constants *k*_classical_ and equilibrium values *X*^0^. The latter are based on the reference structures, mostly a crystal structure. The force constants are 100 kcal·mol^–1^·Å^–2^ for bonds, 20 kcal·mol^–1^ for angles, and 1 kcal·mol^–1^ for dihedral angles. Native contacts are modeled using a 12–10 potential, where *A*_*ij*_ is the distance of the Cα atoms of residues *i* and *j* in the reference; *k*_*ij*_ is 1 kcal·mol^–1^. Nonnative contacts are prevented with an excluded volume potential if they are more than three residues apart in sequence space. σ_*ij*_ is the repulsion radius, and *k*_excluded volume_ is set to 1 kcal·mol^–1^. The repulsion distance *r*_*ij*_ is 2.0 Å for protein beads and 3.7 Å for DNA beads. If the DNA model has a target-site potential, the repulsion distance for DNA is reduced to 3.0 Å to allow contacts between protein and DNA. The protein residues lysine, arginine, and histidine, if protonated, carry a charge of +1; glutamate and aspartate as well as the DNA backbone phosphates carry a charge of −1. The interaction of charges is modeled using the Debye–Hückel equation (eq 5).^44,48^*B*(κ), the ion dependency, is approximated to 1 for diluted solutions and −κ, the Debye screening length, depends on the ionic strength *c*_i_.^49^*K*_Coulomb_ is set to 332 kcal·mol^–1^. For the simulations in this work, we used ionic strengths *c*_i_ ranging from 0.01 to 0.06 M and a dielectric constant ε of 70. We note that, due to the coarse-grained representation of the protein and DNA, the screening effect by salt is shifted to lower concentration values than expected from atomistic representation of the molecules.
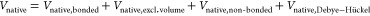
1
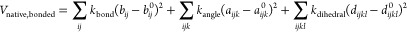
2
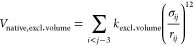
3
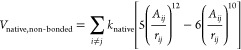
4
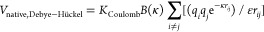
5

The diffusion of the protein on the DNA was sampled using Langevin dynamics with a friction coefficient γ of 0.01.^48−50^

### Parameters of the Recognition Conformation

The starting structure of the dimeric LacI repressor in the recognition conformation is based on the crystal structure with PDB ID 1EFA.^40^ More details of the model can be found in work by Liao and co-workers^43^ and in the Supporting Information.

### Parameters of the Search Conformation

The NMR structure with the PDB ID 1OSL contains 20 modes of a nonspecifically bound artificial dimer of the LacI DBD (Figure S1B).^51^ The two DNA-binding domains are connected by a disulfide bridge between two cysteines at position 52 in place of valine in the wild-type protein (Figure S1C). We identified a conformation of the hinge region according to the quality of sliding trajectories in short test simulations (Table S1). Finally, we obtained the full-length dimer using MODELER^52^ by connecting the ends of the hinge regions, defined at serine 61, to the core of the structure with PDB ID 1EFA (Figure 1C).^40^ In the final CG model, residues in the hinge regions were free to move relative to each other by lowering the force constants of nonbonded interactions and the dihedral potentials. The exact parameters for the search model are contained in the Supporting Information.

### DNA Models

The 100 bp generic, straight B-DNA was constructed using Web 3DNA 2.0.^53^ The bound DNA in the crystal structure (PDB ID 1EFA) was used to design the coarse-grained representation of bent, A-type DNA. A target-site potential was introduced into the coarse-grained model (Table S2). This potential has the form of eq 4 and is based on the bonds between protein and DNA in the crystal structure.^40^

### Coarse-Grained Simulations

The box dimensions, in angstroms, span from −240 to 240 for the *x*- and *y*-coordinates and from −225 to 225 for the *z*-coordinate. The DNA is centered at the middle of the simulation box and aligned with the *z*-axis. For studies of facilitated diffusion, only straight DNA has been used. This should be the most common form of the 100 bp long B-DNA that is about 330 Å long as the persistence length of DNA is greater than 400 Å.^54^ The bent DNA was centered in the middle of the simulation box, and the DNA molecule was oriented along the *z*-axis.

To study the one- and three-dimensional diffusion of LacI, we simulated the monomer and DBD dimer in five replicas for 10^8^ MD steps. The full-length dimer was simulated for 10^8^ MD steps in eight replicas for the lower four salt concentrations and in five replicas for two higher salt concentrations that are likely not relevant in the cellular environment as they fail to reproduce experimental values. The 10^8^ MD steps correspond to 5 ms (assuming a step size of 50 ps).

Furthermore, we ran four systems with the DNA containing a target-site potential (Table S2). These were combinations of search and recognition conformations of the protein with straight or bent conformations of specific DNA, respectively. These simulations have a length of 5 × 10^7^ steps for each of the five replicas.

### Trajectory Analysis

We designed an analysis method that could classify 3D and 1D diffusion, where 1D diffusion is further subdivided into helical groove tracking, while the DBP recognition region is aligned to the major groove, and hopping. We obtained data for the center of mass (COM) of both recognition regions in the dimeric DNA binding domain. The data contains a projection of the recognition helix COM on the DNA axis, the *z*-axis of the coordinate system *Z*, the distance of the helix from the DNA axis *d*, and the rotational angle φ obtained from eq 6. The angle is measured between vectors *v̅* and *v̅*_0_, which contain the *x* and *y* positions of the recognition helix COM and the reference point (0,1), respectively. For analysis of the trajectory, we first filtered out 3D diffusion using a cutoff distance *R*_c_ of *d* = 32 Å.^44^ To distinguish groove tracking versus hopping, we only used intervals of minimum 100 MD steps (≈5 ns) of 1D diffusion. Groove tracking was identified by checking if the position of the center of mass of the recognition helix fluctuated by not more than 1.5 bp from the center of the major groove. The periods between groove tracking events, the recognition region outside the major groove, and not in 3D diffusion were counted as hopping events (Figure S2A). The major groove center was in turn defined by relating a specific rotational angle φ to a specific position *Z* along the DNA (Figure S2B). There are short events that add noise to the output of the analysis, so we added some lower bounds. Concretely, hopping events that were less than 1 bp long were counted as parts of groove tracking. Similarly, if groove tracking lasted less than 0.5 ns, it was counted as hopping. Finally, we calculated the average time and the average distance LacI traveled in each type of one-dimensional diffusion as well as the frequency of hopping based on this analysis.
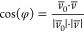
6

### Measuring Translational and Rotational Mean Square Displacements

The mean square displacement (MSD) was calculated and translational and rotational diffusion coefficients were determined from the slopes as described in earlier work.^44^ The translational and rotational MSDs were obtained for position *Z* and angle φ using eqs 7 and 8 with τ in the range from 1 to 200 frames. The diffusion coefficient has been calculated from MSD analysis for τ values between 50 and 200 frames (2.5–10.0 μs). The pitch *p* was obtained using eq 9.
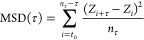
7
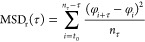
8
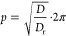
9

### Estimating the Contribution of Hopping to Facilitated Diffusion by LacI

We estimate the contribution of hopping to the overall 1D diffusion with eq 7 and compare it to the purely helical groove tracking *D*_helix_. The 1D diffusion coefficient *D*_1D_ is an indicator of the diffusion rate by combined groove tracking and hopping using the hopping distance *x*_hop_ and the hopping frequency *k*_hop_ to estimate the increase in rate due to hopping.
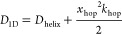
10

## Results and Discussion

It has been suggested in theoretical^39,55−58^ as well as in structural studies^42,51,59^ that the folding of the hinge region into an α helix is crucial for the specific binding of the LacI transcription factor to its operator site. Whereas the structure of the hinge region in the specific complex is known, its native structure in the dimeric full-length LacI during facilitated diffusion has not been reported besides in an NMR study of the truncated DBD.^32,36,42^ To obtain the NMR structures of the nonspecific complex, the core domain of the protein was removed by truncation of the protein right after the hinge helices and a mutation, V52C, was introduced to ensure the dimerization of the LacI DBD by the formation of a disulfide bridge (Figure S1C). Here, we study a recognition conformation (Figure 1B) with helical hinge regions and a search conformation with flexible hinge regions (Figure 1C) using coarse-grained molecular dynamics simulations of facilitated diffusion and binding to understand the function of each of these two conformations. The terms “recognition conformation” and “search conformation” were adopted from earlier work on this topic based on their respective functions to either search the DNA or bind the specific sequence with high affinity.^24^ We first study different truncated models of LacI to examine the role of dimerization and the role of the core domain during facilitated diffusion. Then, we characterize the differences in the facilitated diffusion performed by the different models at increasing salt concentrations. The salt parameter scales the strength of the electrostatic interaction between LacI and DNA. We estimate the average distance traveled, the average duration of hopping and groove tracking, and, last, the electrostatic energy and the extent of formed specific interaction at a DNA target site that is either straight or bent. We aim to clarify if the two conformational states are exclusive to each stage of the binding mechanism. For example, can the recognition conformation (i.e., with helical hinge regions) that was found to bind bent DNA also bind straight DNA and be involved in the search process and can the search conformation (i.e., with flexible hinge loops) interact with bent DNA?

### The Full-Length Representation of LacI Is Necessary to Study Its Binding to DNA

The main difference between the two experimentally identified reference conformations of LacI, the crystal and NMR structures with PDB IDs 1EFA and 1OSL respectively, is the structure of the hinge region. To confirm this, we compared the closest distances between the residues of the transcription factor’s DBD and the DNA backbone. The comparison between the recognition and search conformations shows that the main differences are indeed located in the hinge region (Figure S1A). Furthermore, the distances of the hinge region in the search conformation of LacI show a large standard deviation for the 20 modes included in the NMR structure (Figure S1A,B) which motivated our choice to allow more flexibility in this region as described in the Supporting Information.

We studied the interaction and dynamics of six LacI variants with straight, nonspecific DNA. Three variants of LacI were based on the recognition conformation, and the other three were based on the search conformation. In each case, the three variants were one monomeric form and two dimeric forms of LacI. In one dimeric form, the core domains were excluded, while in the other the full-length transcription factor was studied. The justification for the elimination of the core domain when studying LacI’s interaction with DNA is twofold. First, it does not have direct interactions with DNA.^55,60^ Second, its conformation is retained between apo and DNA-bound LacI.^43^ It has therefore often been excluded from simulations of both LacI’s sliding mechanisms and its conformational transitions to save computational resources.^55,58,60,61^ That is why, as one of the first steps in our study, we tested if the core domain has an effect of LacI’s interaction with DNA.

To visualize how LacI explores the DNA while linearly diffusing, the position of the center of mass of the recognition region along the DNA axis (i.e., *Z*-axis) and the rotational angle, φ, around this axis were plotted. We focus on the recognition helix because this region contains residues that form specific contacts to base edges in the major groove and are therefore expected to follow the major groove while the protein performs groove tracking in search of the specific site.^10,12,62^ The recognition helix is located in the center of each DBD (Figure S1C).

**Figure 2:**
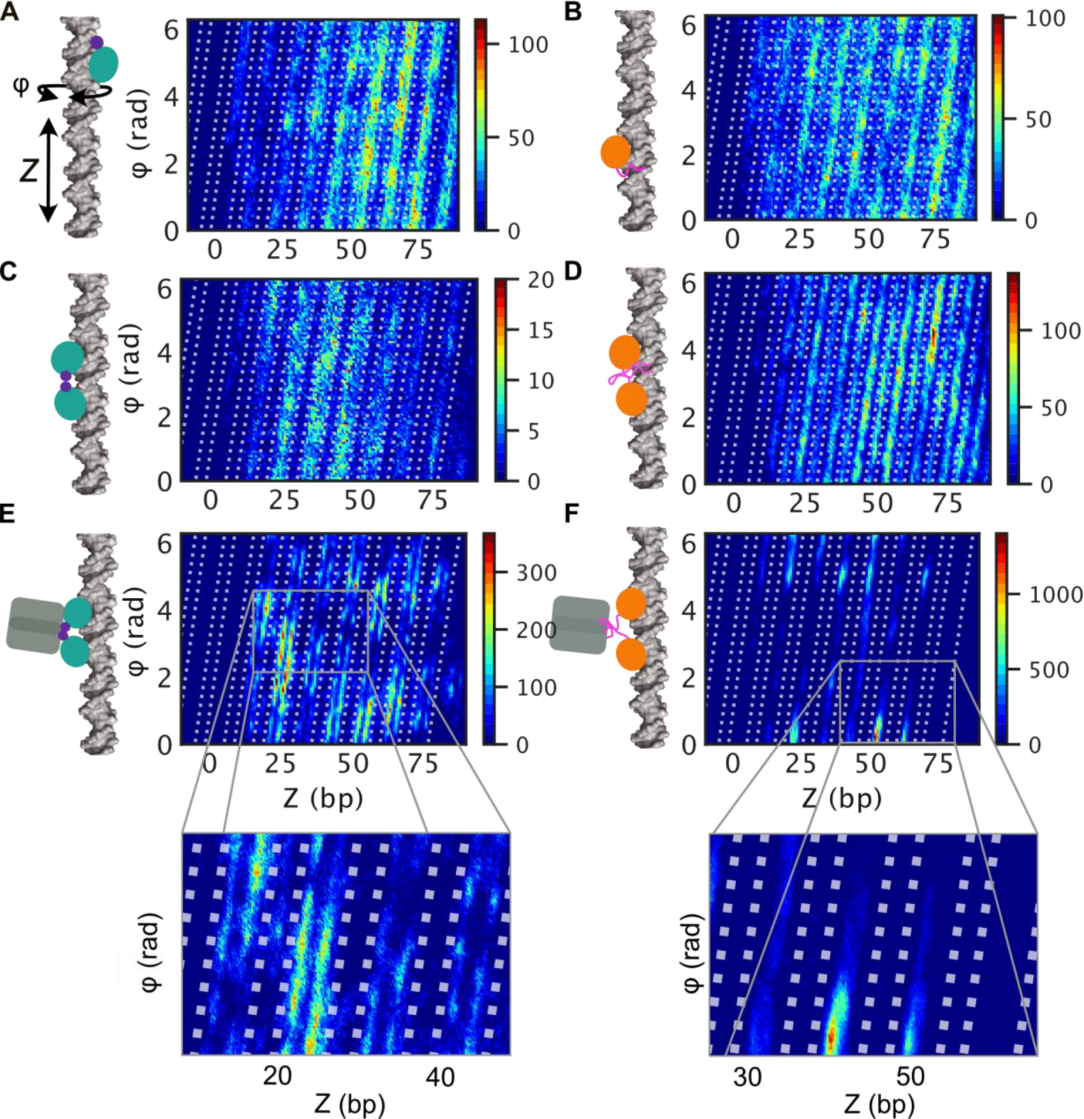
Linear diffusion of variants of LacI along linear DNA studied using coarse-grained simulations. Diffusion of LacI along DNA was studied for the recognition (A, C, and E) and search (B, D, and F) conformations. Each conformation was studied in three variants: monomeric DBD (A and B), dimeric DBD (C and D), and full-length LacI (E and F). The coarse-grained simulations follow the linear diffusion of the *lac* repressor on a 100 bp long coarse-grained DNA. Each panel depicts the translational movement along *Z* and the angular movement around the DNA (φ) collected for each variant of LacI in eight independent simulations, each comprising 10^8^ steps. The plots highlight the areas of DNA that were sampled by the recognition region of the corresponding variant of LacI. The dotted lines in the scatter plots mark the DNA backbones based on the definition in Figure S2. The figure shows results from simulations with salt concentration of 0.02 M. Plots showing the distributions for four different salt concentrations (0.01, 0.02, 0.03, and 0.04 M) are shown in Figures S3 and S4.

Our trajectory analysis shows that monomeric LacI diffuses along the major groove of nonspecific DNA at low salt concentrations (0.01 M) but the interaction is lost when this salt concentration increases (Figure S5B). At a salt concentration of 0.02 M, shown in Figure 2A,B, the monomer does not track the major groove faithfully, a behavior that is more pronounced for the search conformation (Figure 2B). The recognition conformation shows slightly tighter groove tracking reflected by clearly visible traces aligned with the major groove (Figure 2A). According to visual inspection of the trajectories, this pattern originates from the interaction of the rigid, helical hinge region of the recognition conformation with the minor groove. The interaction between the hinge helix and the minor groove assists the positioning of the adjacent recognition helix in the major groove (Figure 2A). In contrast, the flexible hinge region of the search conformation cannot stabilize the interaction of the recognition region in the major groove (Figure 2B). This observation is relevant for interpreting the pattern that we observed for the diffusion of the full-length recognition conformation later on.

Tighter major groove tracking was observed for the dimeric DBD of the recognition conformation (Figure 2C). For the dimeric DBD of the search conformation, helical groove tracking improves as well. The two conformations display two major differences: First, the search conformation samples both minor and major grooves (Figure 2D). The recognition conformation samples mainly the major groove. Second, the recognition conformation interacts with DNA near the edge of the major groove, whereas the search conformation is restricted to the center of the grooves. LacI is known for helical sliding along DNA^8^ and for establishing specific interactions in the DNA major groove.^62^ We therefore expect the protein’s recognition region to probe for those specific interactions during groove tracking and therefore to sample the major groove center.^10,12^ What we observed for the monomeric and dimeric DBDs, without the core domains, was not consistent with these properties. We compared the different models and their sampling of DNA in a range of salt concentrations (Figures S3 and S4). The results show similar trends, but at higher salt concentrations, especially monomers and dimers without the core domain are much more dissociated from the DNA (Figure S5B).

Following these results for the truncated versions of LacI, we added the core domains to the two LacI conformations. We observed less extensive exploration of the nonspecific DNA via diffusion after adding the core domains (Figure 2E,F), suggesting a tighter interaction with the major groove, particularly for the search conformation (Figure 2F). For the recognition conformation, sampling close to the DNA backbones is even more pronounced for the full-length model than for the isolated dimeric DBD, showing a pattern of two rails around the major groove center (Figure 2E). Interestingly, the difference in sliding dynamics when including the core is much more pronounced for the search conformation (Figure 2D,F vs Figure 2C,E). As demonstrated in the cartoons in Figure 2E,F, we found that the flexible hinges of the full-length search conformation allow the alignment of both DBDs with the DNA major groove and sampling of the center of the major groove. For the recognition conformation with a helical hinge region, one hinge helix interacts with the minor groove when the DNA is straight, as we observed for the monomer earlier (Figure 2A). This interaction results in asymmetry for a dimeric LacI. Only if the DNA is bent can both hinge helices fit into the opened minor groove as shown for the specific complex (Figure 1B) and tight interaction can be established.

The effect of ordered versus disordered hinge regions on facilitated diffusion in the two conformations only becomes obvious after adding the core domains. We also find that, in the case of the search conformation, the core domains play an important role in maintaining the necessary alignment of the recognition region with the major groove during groove tracking, which is demonstrated when comparing the sampled DNA regions by the dimeric DBD with the full-length LacI in Figure 2D,F.

### The Search Conformation Displays Better Sliding Properties Than the Recognition Conformation

**Figure 3:**
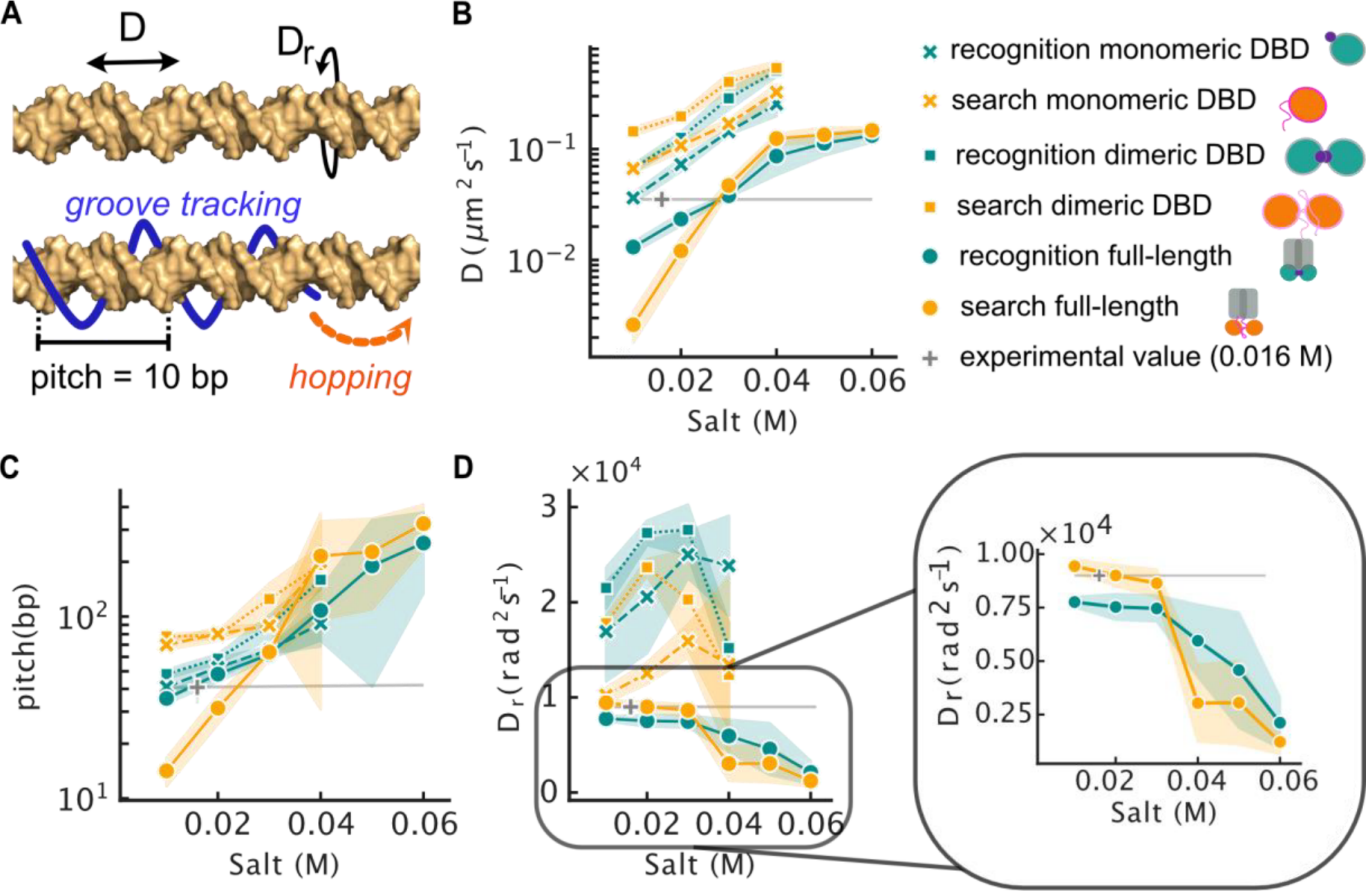
Molecular characteristics of linear diffusion of LacI along DNA: experiments versus computation. (A) Schematic representation of the diffusion dynamics and its molecular characterization. The diffusion is quantified by the diffusion coefficient for linear translocation, *D* (B), the pitch (C), and the rotational diffusion coefficient, *D*_r_ (D). Each plot shows the mean value of the corresponding parameter; the standard deviation is indicated by the shaded region. The pitch is obtained from the two diffusion coefficients via eq 9. The diffusion parameters were measured for the recognition (green) and search (orange) conformations of LacI, in monomeric DBD, dimeric DBD, and dimeric full-length variants. The experimentally measured values of the rotational and translational diffusion coefficients and the pitch at a salt concentration of 0.016 M are shown in gray.

To better quantify the diffusion dynamics of LacI’s recognition and search conformations, we calculated the translational and rotational diffusion coefficients as well as the pitch of facilitated diffusion (Figure 3A, eqs 6–9, Table S3). For the full-length dimeric LacI, the translational diffusion coefficients were generally lower than those for the truncated models (Figure 3B). Figure 3B shows that the search conformation diffuses slower than the recognition conformation at low salt concentration but faster at high salt concentration. For monomeric and dimeric forms, the search conformation diffuses faster or equally fast at all salt concentrations. The pitch is greater for the monomeric and dimeric search conformations when compared to their recognition counterparts (Figure 3C). Since a longer pitch indicates less groove tracking while diffusing, we concluded that monomers and dimers of the search conformation spend less time in the grooves than the monomers and dimers of the recognition conformation. The diffusion properties derived from the CG-MD simulations of LacI on DNA can be compared to corresponding experimental values.^8^ Comparing the values of the diffusion coefficients and pitch suggests that some models are less consistent with the experimental results (Figure 3B–D). The agreement is much better for full-length LacI than for monomeric or dimeric DBD models. This confirms that the core contributes significantly to the properties of the search mechanism.

The computational translational diffusion coefficient of the full-length LacI recognition conformation and the full-length LacI search conformation agree with the corresponding experimental values for salt concentrations of 0.02 and 0.03 M (Figure 3B). The computational pitches of both are in the range of the experimental pitch as well (Figure 3C). Nonetheless, the rotational diffusion coefficient of the search conformation agrees better with the experiments than does the rotational diffusion coefficient of the recognition conformation (Figure 3D). Overall, the computational properties of diffusion for the search conformation of the full-length LacI are in better agreement with the experimental values.

Another interesting characteristic of the full-length LacI in the search conformation is the 10 bp pitch at the lowest salt concentration, indicating diffusion by helical groove tracking only. The recognition conformation does not achieve this close interaction with DNA (Figure 3C). We also observe that the salt concentration has a stronger effect on the full-length search conformation than on the full-length recognition conformation as the diffusion coefficient and pitch increase faster with increasing salt concentration for the search conformation (Figure 3B,C). The rotational diffusion coefficient is initially higher for the search conformation at low salt concentrations before it drops from about 8000 rad^2^ s^–1^ to around 2500 rad^2^ s^–1^, i.e., below the value of the recognition conformation, for salt concentration increasing from 0.03 to 0.04 M (Figure 3D). Again, this indicates that the full-length search conformation is tracking the grooves more faithfully compared to its recognition counterpart until the charge screening loosens the electrostatic interaction and close interaction between protein and DNA can no longer be established. For the more flexible search conformations, charge screening will lead not only to a less tight interaction with DNA but also to higher disorder in the flexible regions that in turn loosen the interaction to DNA additionally. This is not the case for the structured recognition conformation. This shows how flexible regions have a greater scope for different types of interaction, another important argument for the use of disordered regions during molecular search processes. A protein with disordered regions is very sensitive to changes in its environment, such as charge screening in our example. Changing its electrostatic interactions with DNA dramatically changes the search mechanism and therefore the likelihood or rate of specific binding. In a biological system, with a stable salt concentration, such a change in environmental variables could be the concentration of a small molecule that interacts with the protein, such as an inducer like lactose or isopropyl β-d-thiogalactoside in the case of LacI, which may influence its conformational equilibrium.

### One-Dimensional Diffusion of LacI Is Dominated by Hopping Dynamics

**Figure 4:**
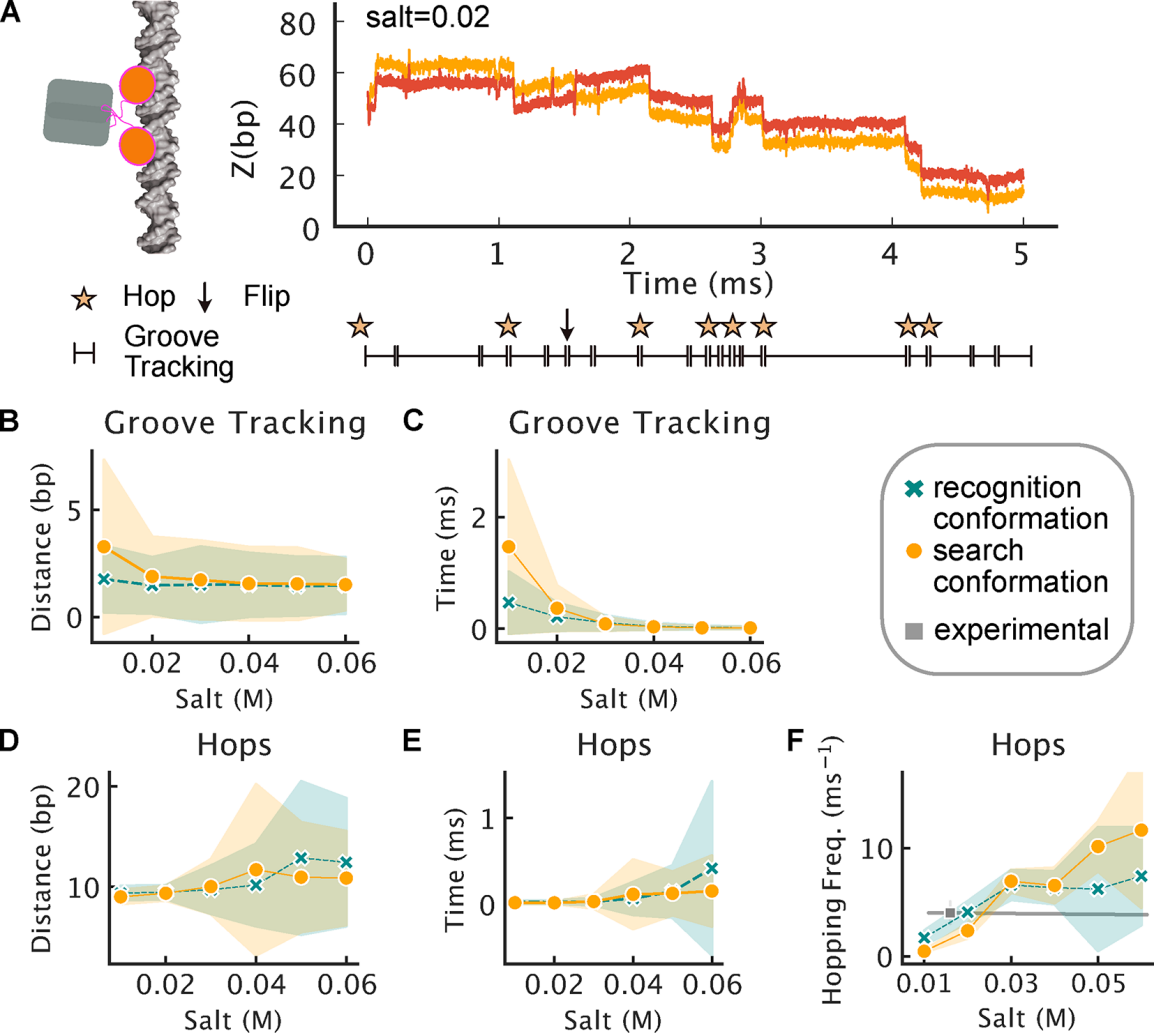
Structural and dynamic properties of LacI diffusion along DNA. (A) Typical trajectory of the full-length search conformation of LacI diffusing along DNA. The locations of the two recognition regions are shown by light and dark orange curves as a function of time. The panel under the plot demonstrates when the analysis algorithm detects groove tracking (line), hops (stars), and flips (arrows). The distances covered during groove tracking and hopping at different salt concentrations are shown in (B) and (D), for the full-length dimeric LacI in the search (orange) and recognition (green) conformations. The respective durations of these events are shown in (C) and (E). (F) Frequencies of hops that are longer than five base pairs. The frequency is compared to the experimental value that is shown in gray.^8^

Next, we quantified the rotation-coupled groove tracking and rotation-decoupled translation (i.e., hopping dynamics) for the full-length dimeric LacI. Figure 4A shows the positioning of the two recognition regions on the DNA for a representative trajectory of the search conformation. As LacI diffuses linearly along the DNA axis (i.e., *Z*-axis), it performs several hops, characterized by large changes in *Z*. After about 1.5 ms, a flipping is observed, where LacI turns 180° around its symmetric axis. This trajectory and the average of all measured groove-tracking events in the simulated trajectories illustrate that the dimeric LacI moves on average about 2 bp during groove tracking that lasts 370 μs for a salt concentrations of 0.02 M (Figure 4B,C, Tables S4 and S5). For a salt concentration of 0.03 M the average length is 1.7 bp and the mean duration is about 90 μs. Increasing the salt concentration, which causes weaker electrostatic interaction between protein and DNA, results in a nearly 4 times shorter duration of groove tracking which is accompanied by a small decrease of scanned DNA (Figure 4B,C). Therefore, temporarily longer interaction with the groove does not necessarily mean that more DNA is explored during groove tracking. The theoretical salt concentration that would model the real system best possibly lies between 0.02 and 0.03 M, and the average duration of groove tracking along 2 bp would therefore lie between 90 and 370 μs. Salt effects on groove tracking are stronger for the search conformation than for the recognition conformation, as we also observed for the diffusion coefficients and the pitch (Figure 3B–D).

Overall, the differences between the parameters of single groove tracking or hopping events performed by the search and recognition conformations of LacI are relatively small. One of the main differences is that the search conformation of LacI performs longer periods of groove tracking that can span more base pairs at 0.01 M salt (Figure 4B). However, these differences vanish for higher salt concentrations which are likely more relevant in the biological context as the comparison to experiments revealed. Hopping events are very similar between both conformations of LacI and last on average about 20 μs, about 4–15 times shorter than groove tracking (Figure 4C,E, Table S5). Hops usually cover around 10 base pairs (Figure 3A,D, Table S5) because LacI unbinds from one position of the major groove and moves rotation decoupled, over the DNA backbones, along the DNA until it finds the next period of the major groove. At higher salt concentration, the hopping duration increases and consequently the distance covered in each hop is longer. With increasing salt concentration, also the hopping frequency increases (Figure 4F). The search conformation has a slightly lower hopping frequency than the recognition conformation at salt concentrations of 0.01 and 0.02 M before it increases more strongly with higher salt, as for the diffusion coefficient. This is again due to the increased flexibility and mobility in the search conformation that follows the screening of electrostatic interactions. The calculated hopping frequency from the CG simulations is in accord with the experimentally determined hopping frequency of 4 hops ms^–1^ (Figure 4F).

Using the results from our trajectory analysis (Table S5) for the search conformation, we assume that the diffusion coefficient for purely helical groove tracking, *D*_helix_, as we found it at a salt concentration of 0.01 M, is 0.0026 μm^2^ s^–1^. To determine the one-dimensional diffusion coefficient *D*_1D_, we used eq 10, which combines *D*_helix_ with the hopping distance *x*_hop_ in Å and the frequency *k*_hop_ in ms^–1^. Using only the mean values reported in Table S5, we estimated *D*_1D_ for salt concentrations 0.02 and 0.03 M in eqs S1 and S2, respectively. For an average hopping length of 9.4 bp multiplied with a base pair step of 3.32 Å length and a mean hopping frequency of 3.24 ms^–1^, we obtained 0.019 μm^2^ s^–1^ at a salt concentration of 0.02 M. At a salt concentration 0.03 M the mean hopping length was determined to be 10.0 bp and the hopping frequency was 6.78 ms^–1^, resulting in 0.04 μm^2^ s^–1^. We note that with pure groove tracking there was a diffusion coefficient of only 0.0026 μm^2^ s^–1^. Therefore, hopping speeds up facilitated diffusion by a factor of at least 7 or considerably higher, depending on the strength of charge screening. This agrees with the finding that groove tracking contributes less than 10% to the overall 1D diffusion coefficient, as shown in previous studies on LacI and other DBPs.^8,63^ That hopping covers considerably longer distances in much less time than groove tracking demonstrates how crucial hopping is for efficient exploration of the DNA.

The comparison of the recognition and search conformations in terms of hopping and groove tracking characteristics shown in Figure 4 demonstrates no significant difference at relevant salt concentrations of 0.02 and 0.03 M. However, based on the analysis of sampled areas, diffusion coefficients, and pitch shown in Figures 2 and 3, we argue that there is a difference between the two. The analysis of hopping and groove tracking as it is demonstrated in Figure 4A shows that we count groove tracking events when the protein remains in one groove, meaning there are no large jumps in the *Z* coordinate, which equals the position of the recognition region along the DNA axis. There is, however, a significant fluctuation in the *Z* position that exceeds what would be expected for groove tracking. These fluctuations are not hopping because the protein quickly returns to groove tracking at the same position. These events and other events that are difficult to classify as either groove tracking or hopping complicate the analysis of groove tracking versus hopping events. Effectively, groove tracking is regularly interrupted by large fluctuations in the *Z*-direction, resulting in shortened records of single groove tracking events. The fluctuation is higher for trajectories with looser association between LacI and the DNA, that is, for the recognition conformation and for both conformations at higher salt concentrations, which consequently show shorter groove tracking events. All in all, it is more straightforward to identify differences in the search properties between the two conformations by comparing either the sampled region or the overall diffusion along and around the DNA by comparing the diffusion coefficients and pitch.

We show in the structural analysis that the disordered helices have an advantage during the search process because they allow alignment of the two recognition helices with the major groove. The more detailed analysis of hopping and groove tracking reveals that, even if they cause a tighter interaction, they do not significantly restrict the agility during search. The effect of flexible protein regions on efficient DNA search was previously illustrated in the context of disordered tails in DNA-binding proteins.^45,46^ For LacI, one effect of flexible regions is to allow asymmetric binding, when only one of the recognition helices binds to the major groove, that can be followed by flipping (Figure 4A) which increases the overall mobility of the DBP. Most importantly, though, the flexible regions allow LacI to perform frequent hopping, which is the main mechanism driving one-dimensional diffusion as shown in our study as well as previously.^8^ All in all, flexible regions are crucial for speeding up exploration of the DNA while allowing a close interaction during groove tracking and therefore recognition of specific sites.

Based on our analysis of groove tracking and hopping, we conclude that the recognition conformation may play a role in the search process, as it exhibits relevant hopping dynamics during facilitated diffusion that agree with experimental values (Figure 4F). In the light of the antenna mechanism in eukaryotic cells,^20^ another state could play a role in the search and recognition mechanism in prokaryotic cells as well. Our model is based on a crystal structure that contains a synthetic operator, OSymL.^40^ This operator has a higher affinity than the three natural operators, O1, O2, and O3, which show structural differences in their specific complexes when it comes to the alignment of the two binding domains with the operator and the bending of the DNA.^37^ Therefore, it is likely that LacI forms a range of different DNA complexes in which different conformational states are stabilized in a sequence-dependent fashion. Such effects are not accessible for studies with our model.

### Recognition Coupled Conformational Changes Are Likely Initiated in the Protein with Disordered Hinge Region When Aligning to the Target Site

**Figure 5:**
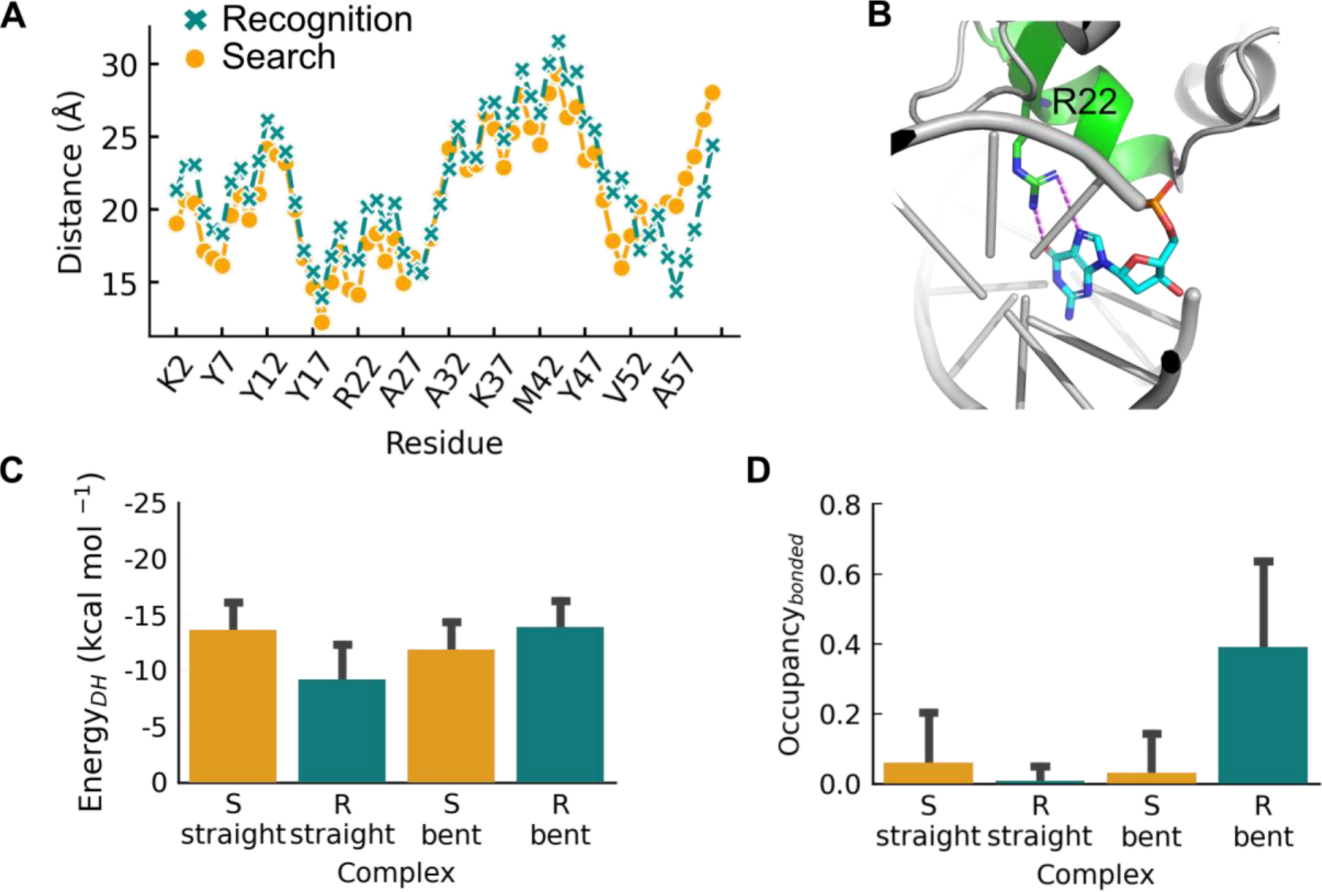
Interplay between conformational changes in LacI and in DNA. (A) Mean distance, *d*, of Cα atoms in the LacI DBD measured from the DNA axis for recognition and search conformations, respectively. The data represents an average over frames recorded during groove tracking performed by full-length LacI. (B) Representative specific interaction between arginine 22 and base G5 in the DNA based on the crystal structure of the specific complex (PDB ID 1EFA). (C) Electrostatic interaction energies of the search and recognition conformations of LacI with either straight or bent specific DNA. (D) Occupancies of specific interactions at the straight or bent target site. The energetics and occupancies of the four complexes between LacI and DNA were obtained from five independent CG simulations with a salt concentration of 0.03 M. The frames for analysis were further selected based on alignment with the target site.

The characterization of facilitated diffusion, as described above, supports that the dimeric LacI in the search conformation scans through nonspecific DNA sequences more efficiently than the recognition conformation. To examine the compatibility of the recognition and search conformations of LacI with straight or bent DNA, the complexes of the two LacI conformations with the two DNA conformations were analyzed. To understand how closely LacI interacts with the DNA major groove during groove tracking, the mean distances between residues in the protein DBD and straight DNA were calculated. The distance profiles show that specifically interacting residues, for example Arg22 in the recognition helix, are closer to the DNA center in the search conformation than in the recognition conformation (Figure 5A). Arg22 is especially mentioned here as it is positioned in the center of the recognition region and forms a strong bidentate interaction in the major groove of the specific complex (Figure 5B) which is known to significantly contribute to the affinity between protein and the operator sequences.^64,65^ The geometrical fit, especially the closeness of specifically interacting protein residues in the complex of the search conformation relative to the recognition conformation with straight DNA supports that the search conformation could recognize the target site on straight DNA. We investigated this in greater detail with simulations of different encounter complexes where LacI can establish specific contacts with the target site.

The encounter complexes were evaluated by measuring the strength of the electrostatic interaction and the ability to form specific interactions by calculating the occupancy of the respective contact (see Methods). The complex between bent DNA and the recognition conformation corresponds to the high affinity/specific complex, known from the crystal structure (PDB ID 1EFA). As expected, this complex has favorable electrostatic interaction energy and the most contacts (Figure 5C,D). The search conformation has a more favorable electrostatic interaction energy and is more likely to form specific contacts with straight DNA than with bent DNA (Figure 5C,D). The recognition conformation has less favorable electrostatic interactions (Figure 5C) and nearly no specific interactions on straight DNA (Figure 5D). The complex between the search conformation and bent DNA forms both electrostatic and specific interactions at the target site to a greater extent than the recognition conformation on straight DNA (Figure 5C,D).

Specificity in protein–DNA interactions is complex and cannot be explained by base or shape readout models alone.^10^ Specific interaction seems to be constituted by an alignment of favorable electrostatic interactions, hydrogen bond donor–acceptor patterns, π–π interactions, and fitting shapes between both molecules.^12,66−68^ It has been argued that a preorganization of interactions that favor the formation of a transition state might play a role in specific protein–DNA recognition.^23^ This idea can be compared to the role of electrostatic preorganization in enzymes^69^ and is helpful when thinking about the four simulated encounter complexes. We observed that the recognition conformation of LacI does not fit an operator of B-DNA conformation when it comes to electrostatics and specific interactions (Figure 5C,D). With bent DNA instead, it has very favorable interactions, but the DNA bending may demand a considerable energy penalty that has to be paid. For this reason, and because the recognition conformation is unlikely to form for free repressor,^43^ we assumed that the specific complex does not form by the encounter of the recognition conformation and bent DNA. This leaves us with investigating the search conformation and its interaction with different shapes of the operator. We found that the search conformation has a favorable electrostatic energy with straight DNA with values close to that measured for the recognition conformation on bent DNA (Figure 5C). Several specific interactions can form as well (Figure 5D). A complex between the search conformation and bent DNA has less favorable electrostatic energy (Figure 5C) and less specific contacts (Figure 5D), but it is more favorable than the complex between the recognition conformation and straight DNA at a salt concentration of 0.03 M, suggesting that the search conformation on bent DNA might serve as more stable intermediate state for the transition from search to recognition than the recognition conformation on straight B-DNA. Figure S6 shows that, at lower salt concentration (0.02 M), the electrostatics of the search conformation on bent DNA and of the recognition conformation on straight DNA are rather similar but that of search conformation on straight DNA is still more favorable. We therefore find that the encounter complex of the search conformation of LacI and straight DNA has more favorable interactions than the other two possible encounter complexes (search conformation on bent DNA and recognition conformation on straight DNA) and is therefore most likely to initiate the formation of the specific complex. The formation of the specific complex will likely require bending of the DNA as the interaction of the search conformation on bent DNA, at least at 0.03 M salt concentration, is favored over the recognition complex on straight DNA. The folding of the helix could occur through a similar mechanism as described by Chu and Munõz for the engrailed homeodomain.^70^ The conformational transitions would occur as the concurrent folding of the hinge region and DNA bending as described by van der Vaart.^56^ The DNA bending is crucial to allowing the accommodation of the two forming helices in the DNA minor groove. The formation of the specific complex would in this case be favored by sequence-dependent DNA bendability.^71^ The role of DNA shape or bendability readout in protein–DNA recognition is well acknowledged,^33,68^ yet their coupling to protein conformational changes is less so. DNA conformational switching as part of recognition has, for example, been discussed for the sex-determining region Y, the TATA-box binding protein, and endonucleases.^71^ We argue that, in the case of LacI, DNA bending is a precondition for the formation of specific and electrostatic interactions between the recognition conformation and the operator. The formation of helices in the hinge region, the transition to the recognition conformation, and DNA bending are coupled processes. They follow the alignment of electrostatic patches and specific interactions between the protein’s recognition region and the DNA major groove while LacI is in the search conformation and the operator is straight. Several other studies support this mechanism. For example, work on solute and salt effects on repressor to operator binding suggests a transition state where the DNA is still straight and the nucleation of the hinge helices has just begun.^39^

## SUMMARY AND CONCLUSION

In this study, two distinct conformations of the *lac* repressor on straight and bent DNA were modeled. We studied the facilitated diffusion on straight DNA in three different forms: a monomeric variant, excluding the core domain, and two dimeric variants (a truncated dimer, excluding the core domains, and a full-length dimer). We found that the core domain has a great influence on the search dynamics, especially for the search conformation with flexible hinge regions. When comparing the two conformations with helical and flexible hinge regions, we found the conformation with flexible hinge regions to be the most suitable for an efficient search of B-DNA, yet we cannot exclude that the recognition conformation (i.e., with a helical hinge) is also partially involved in searching B-DNA. Our model reproduces experimental results for 1D diffusion of LacI on DNA^8^ and provides further insights into the molecular structures during the search. It explains how flexible hinge helices enable tighter interaction with the major groove. We believe that this tight interaction is crucial for recognition of the target site by LacI via hydrogen bond donor and acceptor patterns and via shape-dependent alignment of opposite charges in the two molecules. We also show that screening by ions has a stronger effect on the more flexible search conformation because, first, it interacts tighter with the DNA due to missing steric hindrance and, second, because its internal flexibility is influenced by the charge screening to a greater extent than that of the recognition conformation. In agreement with the experiments, we find that hopping drives the diffusion of LacI on DNA. The flexibility in the hinge region of the search conformation is crucial for groove tracking and hopping, and we emphasize that this structural freedom is key to combining fast exploration, via hopping, with specific recognition, via groove tracking.

In addition to understanding the role of flexible hinge regions in LacI during facilitated diffusion, the CG-MD shows that the interactions between the recognition conformation and straight DNA as well as between the search conformation and bent DNA are weaker than the interaction between the search conformation and straight DNA. Yet, the interactions between the search conformations and bent DNA are more favorable than the interactions between the recognition conformation and B-DNA, suggesting that DNA bending may precede the conformational changes in LacI, as the latter most likely involves a higher energetic barrier. This scenario does not exclude the possibility of coupling between the conformational transition of DNA and that of LacI. This supports a long-standing hypothesis on the LacI binding mechanism.^42,56^

Our work demonstrates the importance of considering the protein as well as the DNA conformation when studying protein–DNA binding and shows that coarse-grained models can assist the study of binding mechanisms. To further quantify the complex mechanism of LacI binding to DNA, atomistic as well as coarse-grained simulations that concentrate on the coupling between the conformational changes of LacI and DNA should and will be employed in future work.

## Notes

The analysis code is available in the GitHub repository (https://github.com/mallu2/CG_analysis.git).^72^ The simulations data are available in DOI: 10.17044/scilifelab.21590394.

## Supporting Information Available

Details on the model; analysis of hopping versus sliding; DNA sampling of all models at all salt concentrations; distances and times of facilitated diffusion; all results from plots in Figures 3 and 4; calculation of the hopping contribution to search; discussion of experimental salt conditions compared with results in this paper (PDF)
