# Supplementary material for: Conformational Change of Transcription Factors from Search to Specific Binding: A lac Repressor Case Study
Source: J Phys Chem B. 2022 Nov 23;126(48):9971–84. doi: 10.1021/acs.jpcb.2c05006 (PMC9743208; doi:10.1021/acs.jpcb.2c05006)
Supplement: Supplementary file 1 — jp2c05006_si_001.pdf [file jp2c05006_si_001.pdf]

Supporting information for:

On the Conformational Change of Transcription Factors from Search to Specific Binding: A Lac Repressor Case Study

Malin Lüking[1], Johan Elf[1] and Yaakov Levy[2,*]

[1] Department of Cell- and Molecular Biology-ICM, Uppsala University, Uppsala, Uppsala county, 751 24, Sweden

[2] Department of Structural Biology, Weizmann Institute of Science, Rehovot, Central District, 76100, Israel

Coarse-grained models of the search and recognition conformation

The search and recognition conformations stem from NMR and crystal structures respectively. Differences in binding can be identified when comparing profiles of distances between Cα-atoms and phosphates of the DNA. Significant differences are located in the hinge regions (residue indexes 52-56).

The input files for the structure based coarse-grained simulation were automatically written based on the experimental structures according to the description in the Material and Methods section. For both conformations, His29 received a charge. This histidine has a theoretical pKa of 7.3 (PropKa[1]). In case of the search-conformation, angles in the flexible regions (K2-P3 and V52-Q60 or residues 1-2 and 51-59) are unified to be at 100 degrees, dihedral angles to 115 degrees. The force constant for dihedrals was lowered to be 0.5 kcal mol^{-1}. For the monomer and dimer, the force constant for the last two residues in the chains, S61 and Q62, was set to 0. Lennard Jones bonds were not considered for a similar region involving residues 1-2 and 53-59. The contacts, that keep the interface stable by an increased force constant (2 kcal mol^{-1}) are: A:50-B:51, A:50-B:52, A:51-B:50, A:51-B:51, A:51-A:52, A:52-B50, A:52-B51, impacting the interactions between R51, V52 and A53 (Fig. S1 C).

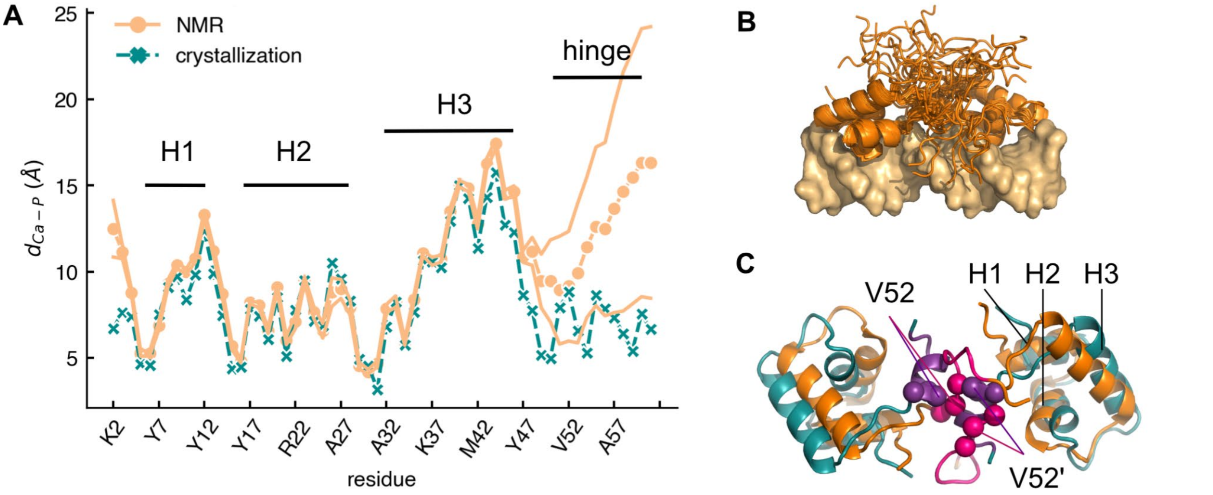

Figure S1. (A) Binding profiles obtained from LacI experimental data used as input for the search (PDB-ID 1OSL[2]) and recognition (PDB-ID 1EFA[3]) conformations. The distances were calculated between Cα atoms and the closest phosphate in the DNA. For the NMR structure mean and standard deviation are shown for the 20 different modes. The location of the four helices in the binding domain are marked with H1, H2 (recognition region), H3 and hinge (hinge loop or helix). (B) NMR-data with 20 different modes. (C) Bird-view of the dimeric DNA-binding domains from crystal (cyan) and NMR (orange) structures, showing residues R51, V52 and A53 in sphere representation, the rest of the protein is shown as cartoon. The hinge loops are colored in pink and the hinge helices in purple. Valine 52 is a cysteine 52 in the NMR-structure, where the disulfide bridge ensures the dimerization.

Table S1. Results of testing different modes of the NMR structure for sliding on the DNA. We tested some of the 20 different modes included in the NMR entry (PDB-ID 1OSL) as input to build models of the search conformation. We ran simulations, 10^7 MD-steps in 5 replicas, using the dimeric model and calculated the correlation of the mean binding profile over the simulation trajectories with the reference binding profile shown in Figure S1 A. We also checked if we could observe sampling of the major groove. The underlined results determined the mode that was chosen to build the final starting structure.

1osl model	Correlation coefficient	*sliding* (major groove sampling)
1	0.8	no
8	0.82	no
9	0.8	no
<u>12</u>	<u>0.82</u>	<u>yes</u>
15	0.8	no
18	0.56	yes
19	-0.42	no

Table S2. Summary of target site parameters used to model the specific interaction between LacI and the operator. The table contains the residue name and index as well as the atom type, chain index and atom index of the atoms in both molecules between which distances have been measured to determine the equilibrium distance used in the native contact potential. Force constants have been determined based on occupancies in all atom simulations[4] and have sometimes been adjusted to the CG-model.

Protein (PDB-ID 1EFA)	DNA	Ca-ID Chain A	P-ID	Ca-ID Chian B	P-ID	force const. (kcal mol^{-1})	D^2 (Å^2) [1] Chain A	D^2 (Å^2) [1] Chain B
Ser16:OH	C3:OP	15	1151	374	851	0,8	33,097	30,980
Tyr17:OH	C5:O	16	1157	375	857	0,2	200,647	203,462
Thr19:OH	C3:OP	18	1151	377	851	0,4	68,079	69,189
Arg22:NH	C3:N/O	21	1151	380	851	0,2	172,397	172,003
Asn25:NH	D11:OP	24	875	383	1175	0,4	54,258	52,766
Ser31:N	D11:OP	30	1148	389	848	0,4	27,134	32,490
Thr34:OH	C2:OP	33	1148	392	848	0,4	52,853	52,853
Tyr47:OH	D10:OP	46	872	405	1172	0,4	99,361	102,495
Asn50:N	D10:OP	49	872	408	1172	0,8	30,008[2]	30,008
Gln54:NH	D:11:OP	53	875	412	1175	0,4	31,025	35,129

[1]The distances were measured between Cα-atoms and phosphate atoms in the crystal structure (PDB-ID 1EFA).
[2]Value copied from Chain B.

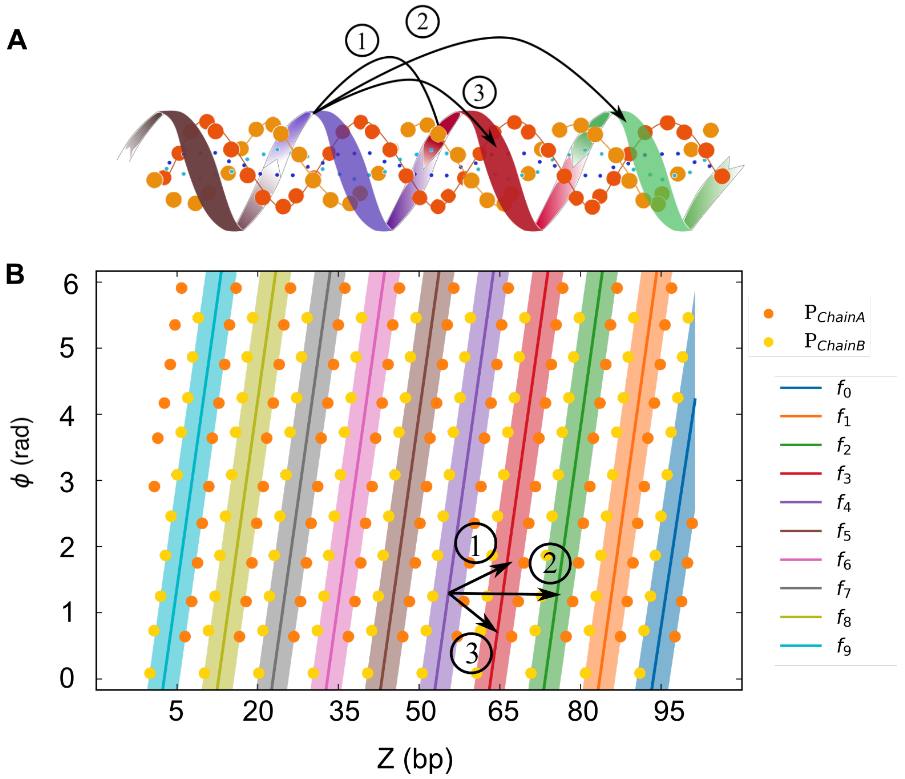

Figure S2. Reference frame for the DNA in the space of the rotational angle φ and position along the DNA *Z*. (A) Sphere representation of the coarse-grained DNA molecule. The backbone phosphate beads are shown in orange and yellow for chain A and B respectively. The bases are shown as small spheres. Four different major groove periods are depicted in colours brown, purple, red and green. Three exemplary hopping trajectories are depicted by arrows. (B) The 2D plot of the DNA surface along the rotational angle φ and position along the DNA *Z*. The DNA phosphate backbone positions in this space are marked by orange and yellow circles for chain A and B and the areas of the major groove are marked by the line along the centre where each line is drawn with a function $f_i(Z) = n_i \cdot Z + m_i = \varphi$ and an area around it that corresponds to 5 bp width.

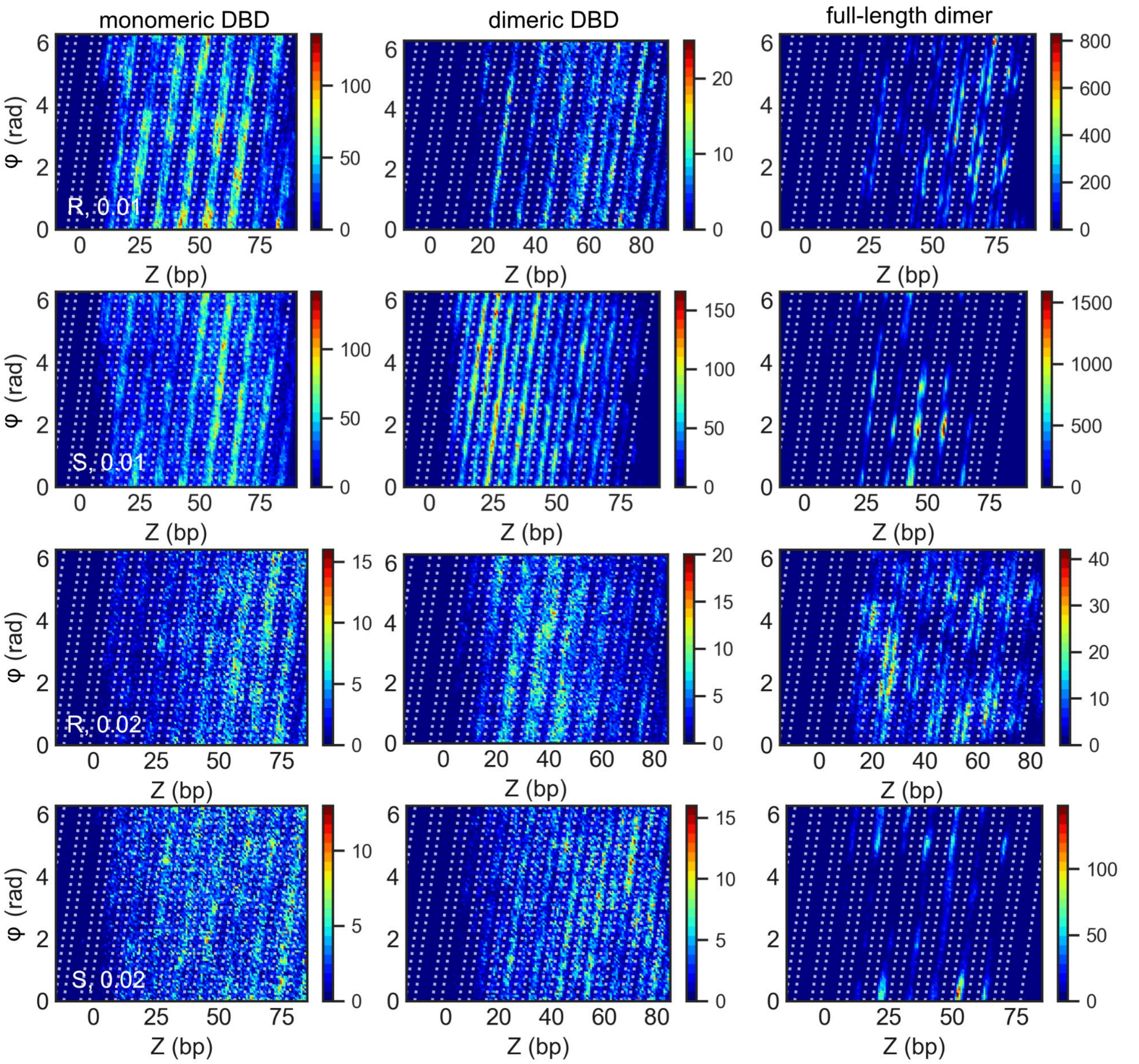

Figure S3. Hex plots of sampled areas by the recognition helices of the recognition (R) and search (S) conformations of LacI at ionic strengths 0.01 and 0.02 M. The colours represent the number of times the recognition region COM has occupied the respective region on the DNA. The dotted lines in the scatter plots mark the DNA backbone.

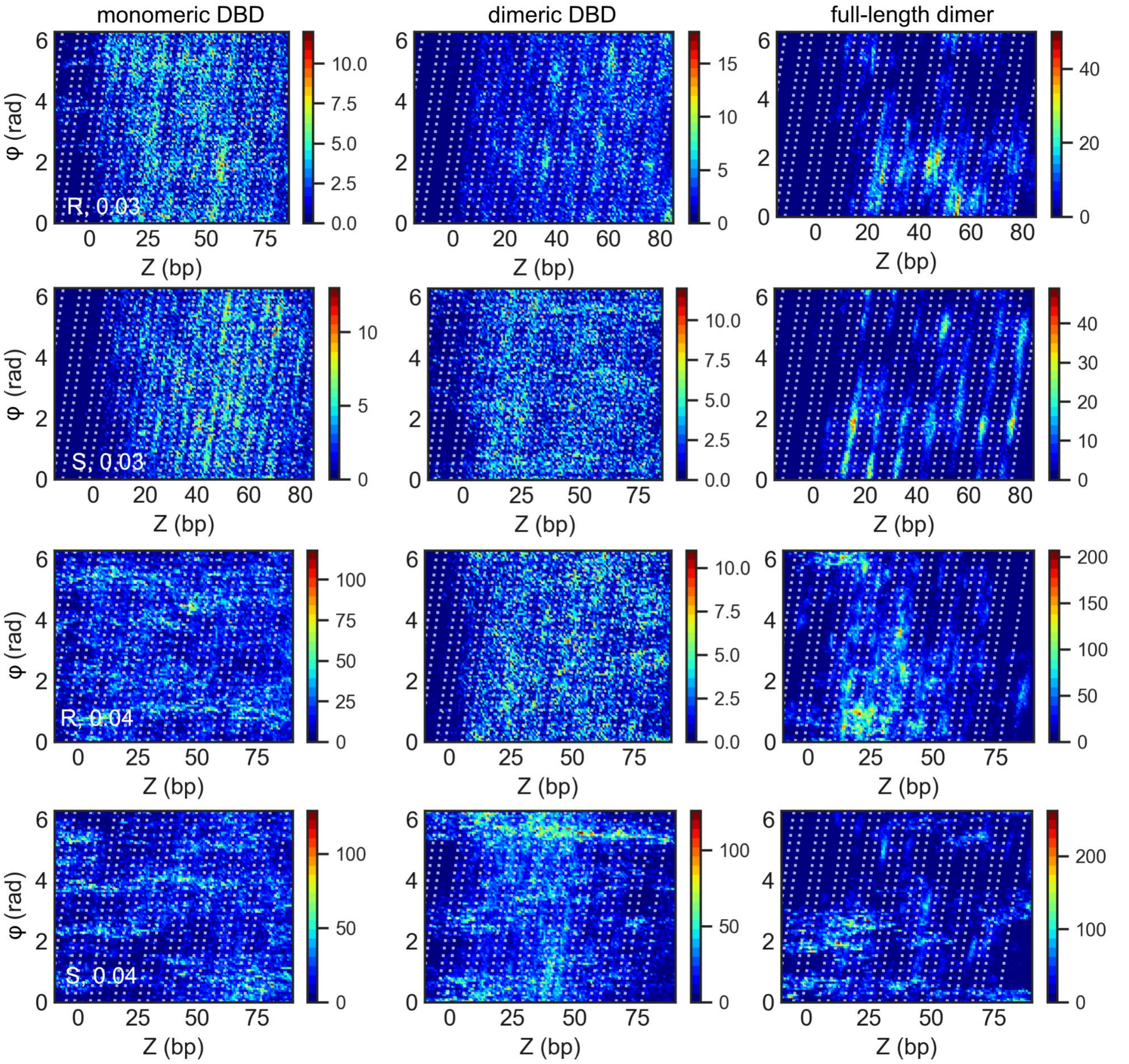

Figure S4. Hex plots of sampled areas by the recognition helices of the recognition (R) and search (S) conformations of LacI at ionic strengths 0.03 and 0.04 M. The colours represent the number of times the recognition region COM has occupied the respective region on the DNA. The dotted lines in the scatter plots mark the DNA backbone.

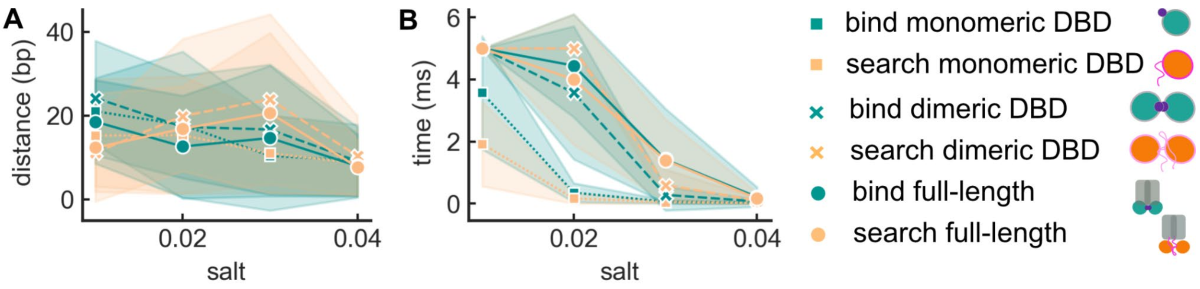

Figure S5. Distance travelled (A) and time spent (B) in facilitated diffusion by the monomeric and dimeric DNA-binding domain as well as the full-length representation of the LacI dimer.

Table S3. Translational and rotational diffusion coefficients D and D_r and pitches at different salt concentrations for search and recognition conformations. The data shows mean and standard deviations for 8 replicas. The low salt values were chosen because the protein is mostly in free diffusion at salt higher than 0.03 M (Fig. S4).

	Recognition conformation			Search conformation		
Salt conc.	D (μm^{2}s^{-1})	D_r (rad^{2}s^{-1})	pitch (bp)	D (μm^{2}s^{-1})	D_r (rad^{2}s^{-1})	pitch (bp)
0.01	0.0131±0.0014	7761±242	35±2	0.0026±0.0008	9478±304	14±3
0.02	0.0234±0.0029	7532±603	48±4	0.0121±0.0036	9003±500	31±6
0.03	0.0380±0.0011	7460±618	62±3	0.0470±0.0057	8640±645	64±5

Table S4. Different parameters determined in CG-simulations of the search state compared to experimental values, if available [5,6]. The table shows mean and standard error of the mean for experimental data and mean and standard deviations for 8 replicas of simulated data with 100 000 frames each.

**Parameter**	**Experiment**	**Simulation (salt=0.02)**	**Simulation (salt=0.03)**
sliding distance	30-40 bp	17 ± 10 bp	21 ± 19 bp
groove tracking distance		2 ± 2 bp	2 ± 2 bp
hopping distance	16 ± 8 bp	9 ± 1 bp	10 ± 3 bp
hopping frequency	4 ± 1 ms^{-1}	3 ± 1 ms^{-1}	7 ± 1 ms^{-1}
pitch	39 ± 9 bp	31 ± 7 bp	64 ± 5 bp

Table S5. Mean and standard deviations for 1D diffusion (sliding), groove tracking and hopping times and distances for LacI in the search conformation at different salt concentrations.

	facilitated (1D) diffusion/*sliding*			
	**time (ms)**		**distance (bp)**	
**salt**	**mean**	**stdev.**	**mean**	**stdev.**
**0.01**	5.0	0.0	12.3	12.9
**0.02**	4.0	2.1	16.9	10.1
**0.03**	1.4	1.3	20.6	19.2
**0.04**	0.2	0.2	7.7	6.6
**0.05**	0.0	0.1	5.8	5.8
**0.06**	0.0	0.0	3.9	3.6

	groove tracking			
	**time (ms)**		**distance (bp)**	
**salt**	**mean**	**stdev.**	**mean**	**stdev.**
**0.01**	1.47	1.56	3.29	4.06
**0.02**	0.37	0.41	1.90	1.86
**0.03**	0.09	0.13	1.74	1.84
**0.04**	0.04	0.05	1.57	1.71
**0.05**	0.02	0.02	1.56	1.69
**0.06**	0.02	0.02	1.52	1.19

			hopping			
	**time (ms)**		**distance (bp)**		**frequency (ms-1)**	
**salt**	**mean**	**stdev.**	**mean**	**stdev.**	**mean**	**stdev.**
**0.01**	0.02	0.02	9.01	0.72	1.10	0.79
**0.02**	0.02	0.01	9.37	0.74	3.24	1.22
**0.03**	0.03	0.08	10.03	2.73	6.78	1.20
**0.04**	0.12	0.40	11.71	8.52	10.33	12.37
**0.05**	0.13	0.25	10.97	5.47	8.42	4.39
**0.06**	0.16	0.41	10.88	4.69	9.55	6.10

Estimating contribution of hopping and groove tracking to the overall facilitate diffusion

We used equation 10 to calculate the translational diffusion coefficient D_{1D}, that describes the mean square displacement by combined groove tracking and hopping. This equation takes the hopping distance x_{hop} and frequency k_{hop} to calculate the mean square displacement by hopping. This way we can compare the speed up due to hopping compared to pure helical groove tracking that goes into the equation by the diffusion coefficient D_{helix}.

Equation S1. Effective diffusion coefficient of LacI on DNA in CGMD simulations at salt concentration 0.02 M.

$$D_{1D}(c = 0.02) = 0.0026\,\mu m^2\,s^{-1} + \frac{(9.37\,bp \cdot 3.32\,Å\,bp^{-1})^2 \cdot 3.24\,ms^{-1}}{2}$$
$$= 0.0026\,\mu m^2\,s^{-1} + 0.016\,\mu m^2 s^{-1} = 0.0186\,\mu m^2 s^{-1}$$

Equation S2. Effective diffusion coefficient of LacI on DNA in CGMD simulations at salt concentration 0.03 M.

$$D_{1D}(c = 0.03) = 0.0026\,\mu m^2\,s^{-1} + \frac{(10.03\,bp \cdot 3.32\,Å\,bp^{-1})^2 \cdot 6.78\,ms^{-1}}{2}$$
$$= 0.0026\,\mu m^2\,s^{-1} + 0.038\,\mu m^2 s^{-1} = 0.0406\,\mu m^2 s^{-1}$$

Notes on experimental salt concentration

The imaging buffer for the single molecule tracking of fluorescently labelled LacI contained 10 mM potassium phosphate pH 7, 1 mM NaCl, 0.1 mM EDTA, 5% (v/v) glycerol, 0.5 mg/ml BSA and 1 mM β-mercaptoethanol [6]. 10 mM potassium phosphate at pH 7 contain 0.0053 M dibasic potassium phosphate and 0.0046 M monobasic potassium phosphate, resulting in a molar concentration of 0.0152 M potassium ions in the buffer. With the additional 1 mM NaCl, the concentration of positively charged ions is 0.0162 M.

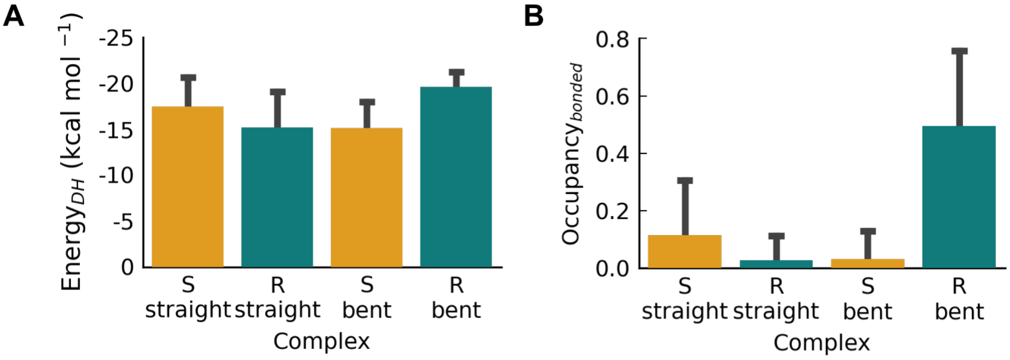

Figure S6. (A) Electrostatic interaction energies of the search and recognition conformations of LacI with either straight or bent, specific DNA. (B) Occupancies of specific interactions at the straight or bent target site. The energetics and occupancies of the four complexes between the LacI and DNA were obtained from five independent CG simulations with a salt concentration of 0.02 M. The frames for analysis were further selected based on alignment with the target site.

References

(1) Olsson, M. H. M.; Søndergaard, C. R.; Rostkowski, M.; Jensen, J. H. PROPKA3: Consistent Treatment of Internal and Surface Residues in Empirical pKa Predictions. *J. Chem. Theory Comput.* **2011**, *7* (2), 525–537.

(2) Kalodimos, C. G.; Biris, N.; Bonvin, A. M. J. J.; Levandoski, M. M.; Guennuegues, M.; Boelens, R.; Kaptein, R. Structure and Flexibility Adaptation in Nonspecific and Specific Protein-DNA Complexes. *Science* **2004**, *305* (5682), 386–389.

(3) Bell, C. E.; Lewis, M. A Closer View of the Conformation of the Lac Repressor Bound to Operator. *Nat. Struct. Biol.* **2000**, *7* (3), 209–214.

(4) Liao, Q.; Lüking, M.; Krüger, D. M.; Deindl, S.; Elf, J.; Kasson, P. M.; Lynn Kamerlin, S. C. Long Time-Scale Atomistic Simulations of the Structure and Dynamics of Transcription Factor-DNA Recognition. *J. Phys. Chem. B* **2019**, *123* (17), 3576–3590.

(5) Mahmutovic, A.; Berg, O. G.; Elf, J. What Matters for Lac Repressor Search in Vivo--Sliding, Hopping, Intersegment Transfer, Crowding on DNA or Recognition? *Nucleic Acids Res.* **2015**, *43* (7), 3454–3464.

(6) Marklund, E.; van Oosten, B.; Mao, G.; Amselem, E.; Kipper, K.; Sabantsev, A.; Emmerich, A.; Globisch, D.; Zheng, X.; Lehmann, L. C.; Berg, O. G.; Johansson, M.; Elf, J.; Deindl, S. DNA Surface Exploration and Operator Bypassing during Target Search. *Nature* **2020**, *583* (7818), 858–861.
